# Review of the effect of 3D medical printing and virtual reality on urology training with ‘MedTRain3DModsim’ Erasmus + European Union Project

**DOI:** 10.3906/sag-1905-73

**Published:** 2019-10-24

**Authors:** İlkan TATAR, Emre HURİ, İlker SELÇUK, Young Lee MOON, Alberto PAOLUZZI, Andreas SKOLARIKOS

**Affiliations:** 1 Department of Anatomy, Faculty of Medicine, Hacettepe University, Ankara Turkey; 2 Department of Urology, Faculty of Medicine, Hacettepe University, Ankara Turkey; 3 Department of Gynecologic-Oncology, Zekai Tahir Burak Research and Educational Hospital, Ankara Turkey; 4 Department of Orthopedics, Chosun University, Chosun, South Korea; 5 Department of Mathematics and Physics, Rome Tre University, Rome Italy; 6 Department of Urology, National Kapodistrian University, Athens Greece

**Keywords:** 3D medical printing, anatomy, virtual reality, urology training

## Abstract

**Background/aim:**

It is necessary to incorporate novel training modalities in medical education, especially in surgical fields, because of the limitations of cadaveric training. Traditional medical education has many drawbacks, such as residency working hour restrictions, patient safety conflicts with the learning needs, and the lack of hands-on workshops. The MedTRain3DModsim Project aimed to produce 3-dimensional (3D) medical printed models, simulations, and innovative applications for every level of medical training using novel worldwide technologies. It was aimed herein to improve the interdisciplinary and transnational approaches, and accumulate existing experience for medical education, postgraduate studies, and specialty training.

**Materials and methods:**

This project focused on models of solid organs and the urinary system, including the kidney, prostate, ureter, and liver. With 3D medical printing, it is possible to produce a body part from inert materials in just a few hours with the standardization of medical 3D modeling.

**Results:**

The target groups of this project included medical students and residents, graduate students from engineering departments who needed medical education and surgical training, and medical researchers interested in health technology or clinical and surgical anatomy.

**Conclusion:**

It was also intended to develop a novel imaging platform for education and training by reevaluating the existing data using new software and 3D modalities. Therefore, it was believed that our methodology could be implemented in all related medical fields.

## 1. Introduction

Surgical education has usually been based on the Halstedian methodology of “see one, do one, teach one”. This methodology depends on the volume of, as well as the access to, patients. The field of surgery covers a wide range of complicated procedures. Teaching or learning with the Halstedian method is a challenge due to the increased public awareness of patient safety. Furthermore, there is the lack of wide-scale availability of materials for learning surgery operations. Thus, there is definitely a need for new solutions for training medical students about surgical operations [1].

Even though cadavers have been used for ages, human dissection has always been an object of controversy due to the religious prejudices and ethical bias raised in civilized societies [2,3]. In addition, cadavers, as a mean of teaching, pose many problems that prevent their widespread use. Those problems include organizational and logistic factors, such as the need for trained personnel, the lack of an efficient number of corpses available for dissection to prevent student overload, the high cost of maintaining dissection labs, and health risks due to prolonged exposure and contact with corpses [2]. Thus, it is necessary to develop novel training modalities in medical education to overcome the limitations of cadaveric training. Traditional medical training has many difficulties, such as residency work hour restrictions, patient safety conflicts, and the lack of hands-on workshops [4].

These educational limitations provide room for the improvement of medical training using digital simulations, 3-dimensional (3D) medical applications (virtual reality (VR) and augmented reality (AR)), and 3D-printed models. Digital simulations and 3D medical applications might never be able to replace clinical experience and hands-on training on cadavers or live cases. Current simulation models may, however, decrease the length of the learning curve without compromising patient safety.

The MedTRain3DModsim Erasmus + European Union Project, which started on October 2016 and completed on October 2018, was led by Hacettepe University in Ankara, Turkey, and partner organizations Chosun University, South Korea; Charles University, Czech Republic; and Rome 3 University, Italy and Hellenic Urological Association, Greece. The full name of the project was ‘Novel Educational Materials in Medical Training with 3D Modeling Application and Simulation Modalities (Virtual Reality and Augmented Reality)’, which was the first project funded by the Turkish National Agency. The aim of the project was to extract and reconstruct 3D realistic anatomical models from computer tomography (CT) (Digital Imaging and Communications in Medicine, DICOM) images with various software packages and print or simulate them in 3D for educational purposes. The project focused on models of solid organs and the urinary system, including the prostate, kidney, ureter, and liver. After having completed the project successfully, this review article was written to present the effects of novel innovative approaches, such as medical 3D-printed models, digital simulations, or virtual reality on medical education and, in particular, surgical urology training. 

## 2. Project design

**Figure 1:**
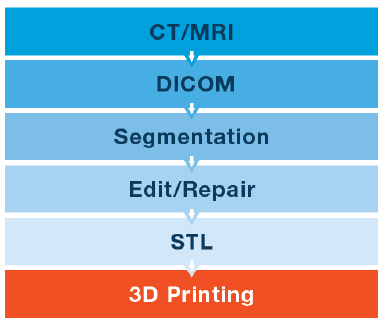
Common generating steps of a 3D model.

Medical 3D simulation technology has developed exciting new solutions and possibilities for medical diagnosis and practice. A common 3D model was used to generate steps for both static biomodels and physical simulators (Figure 1). The processes began with CT or magnetic resonance imaging (MRI) data, from patients or cadavers, which were generated from DICOM files. These were then imported into software programs [(e.g., Materialise Interactive Medical Image Control System (MIMICS); Materialise NV, Leuven, Belgium], where the anatomy was segmented to create the desired anatomic structures. The data was further modified and repaired, wherever needed, with the 3DS MAX and Z-brush 3D model editing tool. The texturing process was performed using Photoshop to express a realistic anatomical texture. Next, polygonal mesh (stereolithography, STL) files were generated for 3D printing (3DP). That data can be used for generating a virtual reality model or a printed model. Following 3DP, the anatomical replicas were used as-is, coated, painted, or dyed. For the physical simulators, 3D-printed replicas were used combined with other materials to imitate tissue, such as silicone, hydrogel. For the urinary system replicas, all of the 3D-printed models, including the lumen, were adaptable to endoscopic urologic devices.

In the project, we used 2 types of 3D-printed models. The first one used 3DP to create molds that were then used to cast anatomic structures in materials that better simulated human tissue. The second one used 3D-printed anatomic replicas, without using a mold, and was directly one-to-one matched to a STL file. The cast materials included silicone, polyurethane, hydrogel, a gelatin/agar mixture, and high-acyl gum.

**Figure 2:**
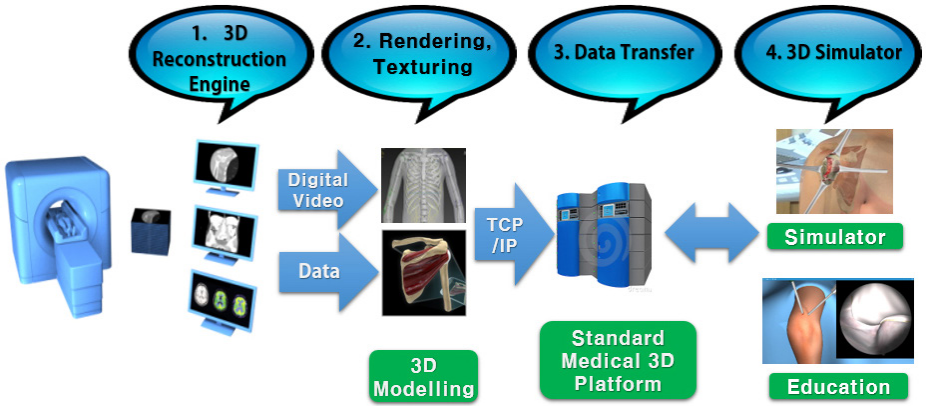
Project’s intellectual output flowchart.

There were 4 intellectual outputs (IOs). The first IO was the reconstruction and 3DP of the customized anatomical models. The second IO was the production of the VR scenarios using these models virtually. The third IO was the standardization process of the 3D modeling and soft tissue printing, and the fourth IO was preparation of the web-based training modules and an application system to preregister the training sessions, videos, lectures and game-based training backgrounds. Specifically, for the first 2 IOs, there was also a general project flow-chart, which is shown in Figure 2 and included the following steps:

· **3D reconstruction engine:** Extraction of CT or MRI data, from patients or cadavers, with a medical imaging device and generating the DICOM files from them with MIMICS,

· **Rendering and texturing: **Masking the area of interest and extracting the STL files of the 3D models with MIMICS and 3D surface rendering, and texturing for the realistic human and surgery tool model with 3DS MAX and Z-Brush,

· **Data transfer:** Transfer 3D models to the standard medical 3D platforms,

· **3D simulator:** Building libraries for the final 3D data with 3D animated surgical movements derived by a 3D controller and converting the 3D data into a VR engine, and creating platform unity 3D.

At the end of the project, 50 pieces of 3D-printed organ models and 11 surgical stations for hands-on training, including 4 VR surgical procedures (game-based training), were successfully produced. In total, 1000 participants and observers were included in the project’s learning & teaching & training (LTT) activities and multiplier event (ME). A group of 290 trainees actively participated in the surgical training using 3D-printed or simulation models. Every participant chose one procedure for the evaluation of each set, separately. However, recurrent applications and participants who had more than one set of training were excluded from the evaluation.

### 3. Products 

The products were classified into 2 groups, as follows:

#### 3.1. Patient-specific CT-reconstructed 3DP models and outputs

**Figure 3:**
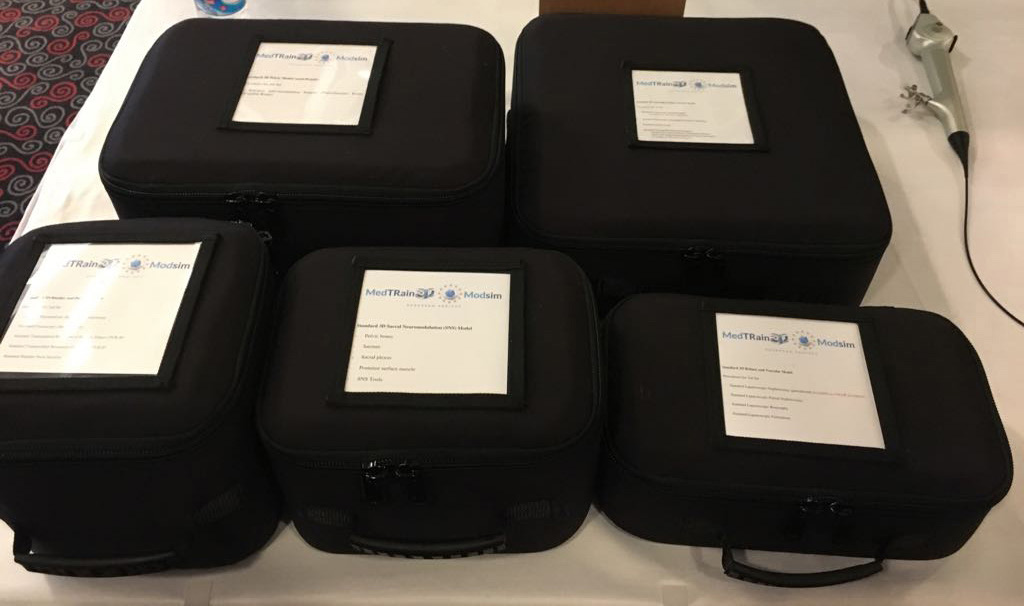
MedTRain3DModsim training boxes for the physical urologic simulators.

**Figure 4:**
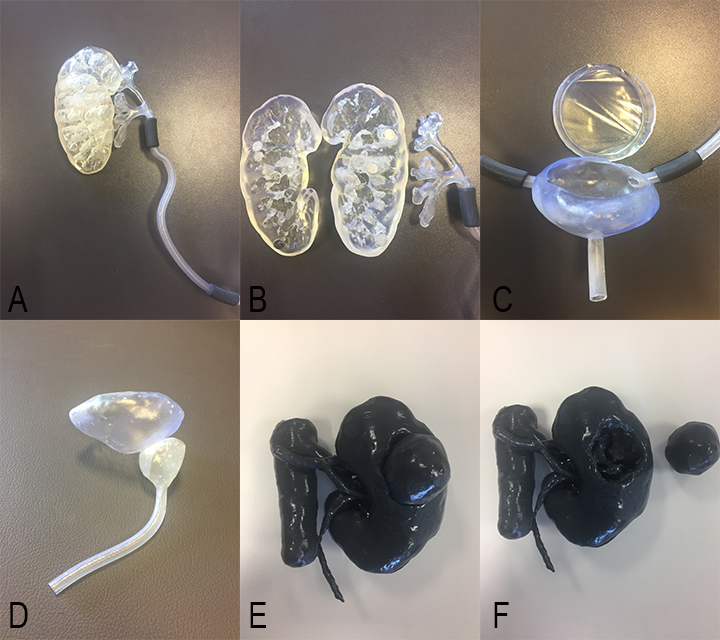
Standard 3D anatomic urinary system model, standard 3D bladder and prostate model, and standard 3D kidney and vascular model. Renal and ureteric (A), renal parenchymal and pelvicalyceal system (B), bladder and urethral (C), and portions of the standard 3D anatomic urinary system model. D. Standard 3D bladder and prostate model. Tumor (E) localized on and excised tumor (F) from the standard 3D kidney and vascular model.

**Figure 5:**
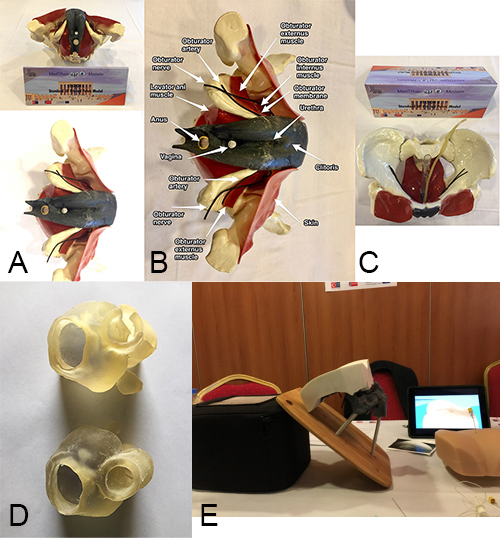
Standard 3D pelvic model (female), standard 3D prostate biomode, and standard 3D sacral neuromodulation (SNS) model. Frontal (upper) and inferior (lower) views (A) of the anatomically labeled (B) and superior view (C) of the standard 3D pelvic model (female). D. Standard 3D prostate biomodel. E. Standard 3D SNS model.

The European Board of Urology (EBU) suggested 14 urologic procedures 3005European_Urology_Residency_Curriculum_by_EBU_-_Web_Form (2019) [Online] Website [accessed 05052019] that need to be assessed for the evaluation of a resident’s skills. Selected were important urologic procedures that were included in the EBU list to create 3D-printed static biomodels or physical simulators for training purposes. The MedTRain3DModsim training boxes (M3DM T-Box), which comprised physical urologic simulators produced by 3DP technology, are shown in Figure 3. A total of 6 sets were prepared as a station. The sets and related surgical procedures are shown in Table 1. The surgical models are shown in Figures 4 and 5. The variables of the 3D-printed models are shown in Table 2. The general assessment of the courses, which were performed using the Likert scale questionnaire, with the median points for “Contribution to your knowledge”, “Eligibility of the physical environment”, “Satisfaction from the organization”, “Education materials”, “Eligibility of the training methods”, “Suitability of the training period”, “Suitability of the content of the education”, and “Satisfaction from training” were 4.21, 4.30, 4.30, 4.12, 4.23, 4.21, 4.35, and 4.30, respectively (Table 3).

**Table 1:** MedTRain3DModsim 3D-printed sets and related surgical procedures.

SET	PROCEDURES
Standard 3D anatomic urinary system model	- Standard cystoscopy (flexible/rigid) (available as VR/AR formation)- Standard retrograde pyelography/double J stenting- Standard ureteroscopy- Standard retrograde intrarenal surgery (inspection of the pelvicaliceal system/relocation of the stone with a basket/disintegration of the stone with a laser) (available as VR/AR formation)
Standard 3D bladder and prostate kodel	- Standard percutaneous suprapubic cystostomy- Standard cystoscopy (flexible/rigid)- Standard transurethral resection of the bladder tumor- Standard transurethral resection of the prostate - Standard bladder neck incision
Standard 3D Kidney and vascular model	- Standard percutaneous nephrostomy- Standard laparoscopic nephrectomy (partial/total) (available as VR/AR formation)- Standard percutaneous nephrolithotomy (C-arm depended)
Standard 3D pelvic model (female)	- Standard antiincontinence surgery (transobturator route, retropubic route) pelvic-perineal detailed anatomy
Standard 3D prostate biomodel	- Only for 3D prostate anatomy training- Diagnosis for prostate cancer/nodule
Standard 3D SNS model	- Sacrum- Sacral plexus- Posterior surface muscle- SNS Tool

**Table 2:** Variables of the 3D-printed models produced during the project. SLA: stereolithography, FDM: fused deposition modeling, Rev Eng: reverse engineering, PLA: polylactic acid.

Models Variables	Kidney	Ureter	Bladder	Prostate +urethra	Pelvicbone	Sacrum	Siliconkidney	Vessel
Image process variable								
Pixel size	0.5 mm	0.5 mm	0.5 mm	0.5 mm	0.5 mm	0.5 mm	N/A	0.5 mm
Slice thickness	1 mm	1 mm	1 mm	1 mm	1 mm	1 mm	N/A	1 mm
Modelling process variable								
Modelling time	8 h	3 h	4 h	3 h	5 h	4 h	12 h	3 h
Anatomic suitability	±1 mm	±1 mm	±1 mm	±1 mm	±1 mm	±1 mm	±1 mm	±1 mm
Production and post process variables								
Production technology	SLA	SLA	SLA	SLA	FDM	FDM	Rev Eng	SLA
Production resolution	0.025	0.025	0.1	0.1	0.2	0.2	0.2	0.1
Production period	16 h	8 h	10 h	9 h	32 h	18 h	36 h	10 h
Post process period	3 h	2 h	1 h	2 h	2 h	2 h	5 h	2 h
Material type (soft/hard)	Resin/hard	Resin/hard	Resin/hard	Resin/hard	PLA/hard	Resin/hard	Silicon/soft	Resin/hard

**Table 3:** General assessment of the models included in the surgical sets.

Variable	Mean	Median	SD	Min	Max
3D anatomic urinary system model usefulness	4.35	4	0.72	3	5
3D anatomic urinary system model realism	3.91	4	0.92	2	5
3D anatomic urinary system model overall	4.26	4	0.79	3	5
3D bladder and prostate model usefulness	4.02	4	0.74	3	5
3D bladder and prostate model realism	3.72	4	0.82	2	5
3D bladder and prostate model overall	4.05	4	0.75	3	5
3D kidney and vascular model usefulness	4.24	4	0.83	2	5
3D kidney and vascular model realism	4.00	4	1.00	2	5
3D kidney and vascular model overall	4.18	4	0.88	2	5
3D pelvic model usefulness	4.26	4	0.72	3	5
3D pelvic model realism	3.95	4	0.81	2	5
3D pelvic model overall	4.26	4	0.69	3	5
3D prostate biomodel usefulness	4.33	5	0.77	3	5
3D prostate biomodel realism	3.91	4	0.67	3	5
3D prostate biomodel overall	4.09	4	0.72	3	5
3D sacral neuromodulation model usefulness	4.49	5	0.70	3	5
3D sacral neuromodulation model realism	4.26	5	0.90	2	5
3D sacral neuromodulation model overall	4.49	5	0.73	3	5
3D VR cystoscopy model usefulness	4.36	4	0.69	3	5
3D VR cystoscopy model realism	4.19	4	0.80	3	5
3D VR cystoscopy model overall	4.43	4	0.59	3	5
3D VR retrograde intrarenal stone surgery model usefulness	4.07	4	0.88	2	5
3D VR retrograde intrarenal stone surgery model realism	4.12	4	0.95	2	5
3D VR retrograde intrarenal stone surgery model overall	4.12	4	0.90	2	5
3D VR laparoscopic nephrectomy model usefulness	4.02	4	1.03	2	5
3D VR laparoscopic nephrectomy model realism	3.74	4	1.09	2	5
3D VR laparoscopic nephrectomy model overall	3.95	4	1.06	2	5
3D VR liver surgery corrosion casting usefulness	4.32	4	0.81	2	5
3D VR liver surgery corrosion casting realism	4.07	4	1.05	2	5
3D VR liver surgery corrosion casting overall	4.18	4	0.86	2	5

#### 3.2. Patient-specific CT-reconstructed 3D VR simulators 

There were 4 simulators as the products of the project: **standard cystoscopy simulator, standard retrograde intrarenal surgery (kidney stone treatment) simulator, laparoscopic nephrectomy simulator, and CatCraft game-based VR training module.**

#### 3.2.1. Standard cystoscopy and standard retrograde intrarenal surgery (kidney stone treatment) simulators 

**Figure 6:**
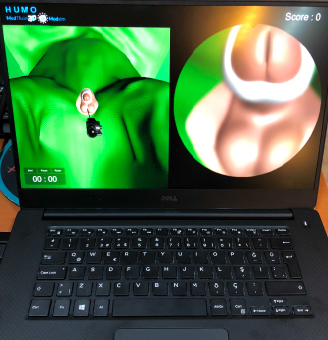
Screenshot ofthe standard cystoscopy and standard retrograde intrarenal surgery (kidney stone treatment) simulators.

The surgical scenario related with these simulators (screenshot from the beginning of the scenario is seen in Figure 6) was that the students could hold cystoscopy or ureterorenoscopy virtually and could control it without haptic feedback. The anatomic landmarks from the urethral meatus to the pelvicalyceal system were objectively classified to teach stepwise anatomy, in addition to a scoring system and time for measurement training session. The total score for retrograde intrarenal surgery and stone fragmentation was divided into 5 parts for 50 point as shown below:

Urethral exposure (10 points), 

Right ureteral orifice exposure (10 points), 

Ureteral complete exposure (10 points), 

Intrarenal exposure (10 points), 

and stone fragmentation (10 points), with virtual endoscopic instruments. Virtual cystoscopy provided the chance to explore intravesical anatomy and pathology without any borders or limits. 

#### 3.2.2. Laparoscopic nephrectomy simulator

**Figure 7:**
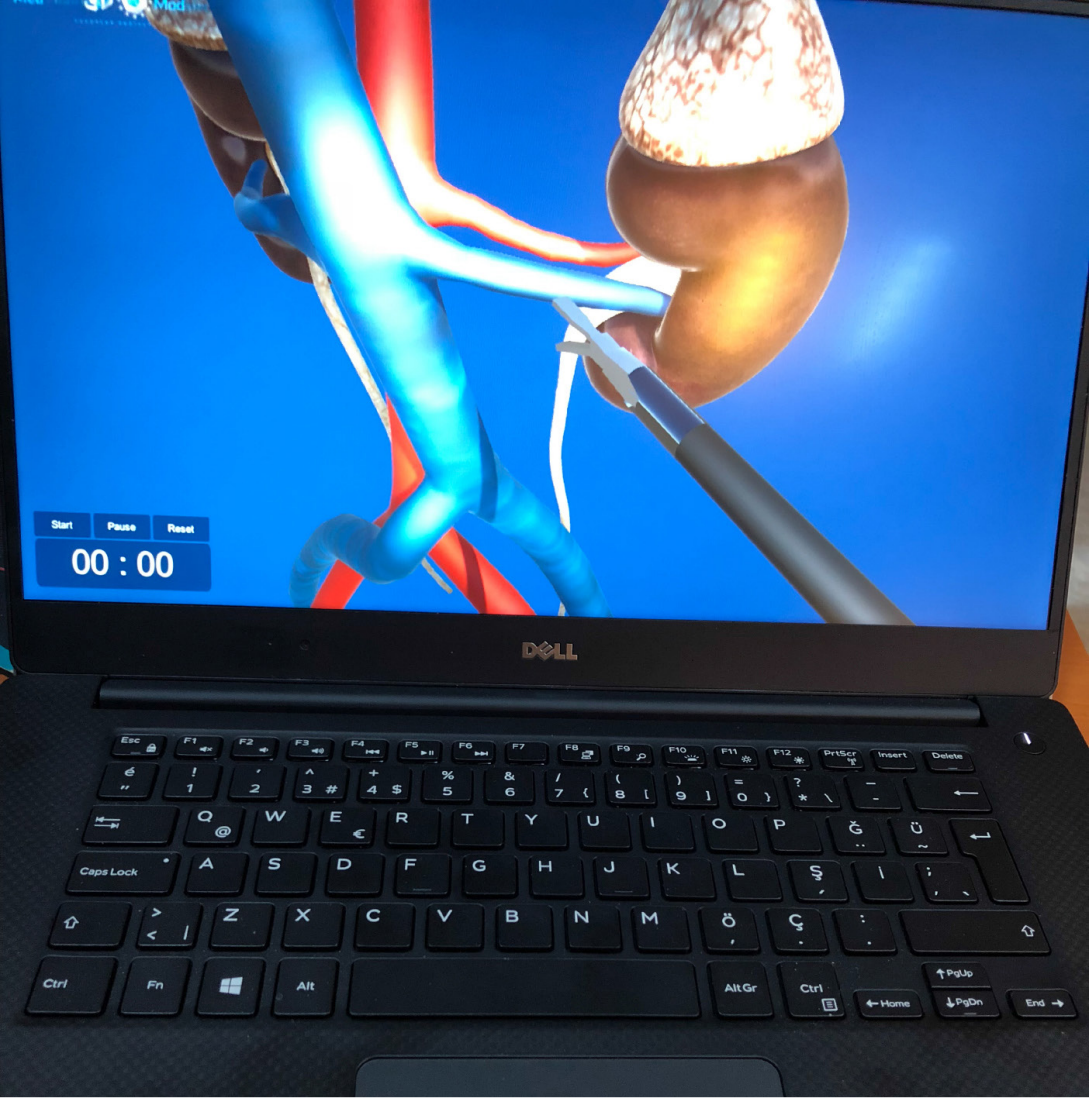
Screenshot of the laparoscopic nephrectomy simulator.

Similar to the previous one, in this simulator, according to the surgical scenario, the student holds a laparoscopic dissector, scissors, and clip virtually, and controls them without haptic feedback. The laparoscopic nephrectomy surgical procedure was objectively classified according to the anatomical stepwise approach. The virtual steps were planned in alignment with the real surgical steps. The steps were: renal artery clipping (with 3 clips), renal vein clipping (with 3 clips), ureteral dissection (with 3 clips), adrenal gland dissection, and removal of the kidney from the monitor. The time was measured for evaluation of the trainee’s skills based on their anatomy and virtual surgical skills (screenshot of it is seen in Figure 7).

#### 3.2.3. CatCraft game-based VR training module

**Figure 8:**
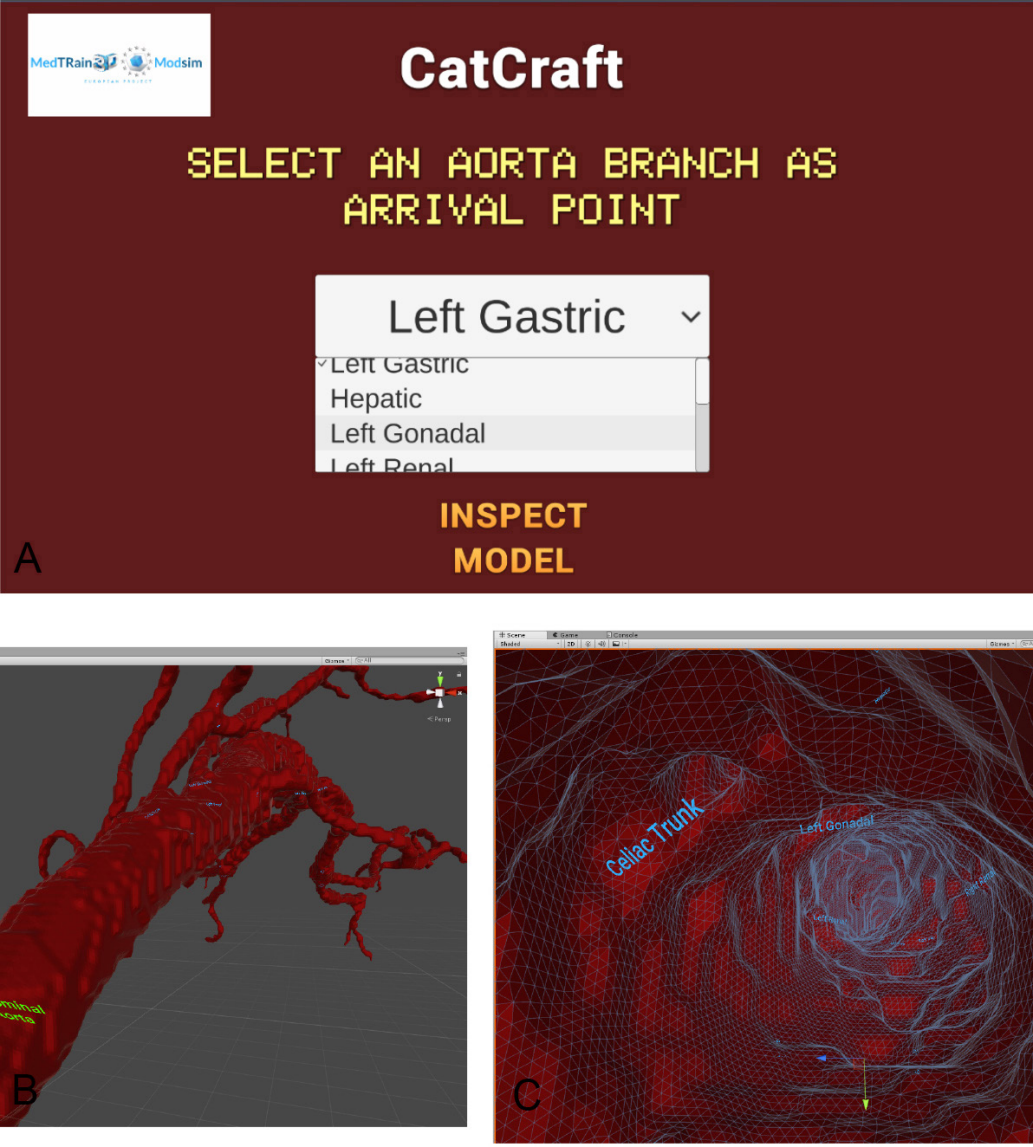
Screenshots of the Catcraft simulator. A) Main menu of the Catcraft simulator. Screenshots from outside (B) and inside (C) of the abdominal aorta.

Within the CatCraft (Figure 8) simulator, the student had the opportunity to navigate inside of a 3D model of the abdominal aorta, which was extracted from real CT scans, with the goal of reaching one of its branches. Thus, the game presented 2 parallel challenges for the players: one was to recall the anatomical structure of the aorta in order to identify correctly the branch to go through, the other was to use the navigation commands to safely reach their destination, by avoiding the walls of the artery and within the shortest possible time. In particular, the students faced a task that tested their eye-hand coordination, by having to cross narrow passages at a high travel speed.

## 4. Discussion

Simulation has become widely accepted as a supplementary method of training. Within urology, the largest number of procedure-specific models and subsequent validation studies has been carried out in the field of endourology. Within the available modalities, VR simulators are the most commonly used for endourology and robotic surgery training, the former also employing many high-fidelity benchmark models. Smaller dry-lab and ex vivo animal models have been used for laparoscopic and robotic training, whereas live animals and human cadavers are widely used for full procedural training. Newer concepts such as AR models and patient-specific simulators have also been introduced [5]. Recently, the effectiveness of various types of simulations was indicated by many authors in the subdivisions of urological surgery training, including urolithiasis [6], and the stone treatment procedure [7], prostate surgery [8], transurethral surgery [9], ureteroscopy [10], percutaneous renal access (PCA) [11], and pediatric urological surgery [12].

Additive manufacturing, or 3D printing (3DP), as it is commonly known, is a process used to create 3D objects from computer-aided designs (CAD). Using sophisticated software, the CAD image files are graphically sliced into successive 2D layers representing the entire 3D object. Processing the CAD images, 3D printers assemble the 3D object layer-by-layer from an array of assorted materials [13]. 3DP was invented by Charles Hull in 1986. The advent of 3DP technology has enabled the creation of a tangible and complex 3D object that goes beyond a simple 3D-shaded visualization on a flat monitor. Since the early 2000s, 3DP machines have been used only for hard tissue applications [14]. The potential applications of 3DP in clinical medicine are numerous. It can allow physicians to create patient-specific models of pathology with such precise anatomic detail that it facilitates preprocedural planning prior to treatments. 3DP can also serve as an important teaching tool and training adjunct in medical education, not only for medical students and residents, but also in the counseling of patients and their families with regard to disease management and procedural description. Finally, 3DP can allow for the creation of bioprinted cells for the testing and development of novel medications or targeted agents, to better replicate its potential use and efficacy in actual patients [15]. 

3DP is an evolving technology that enables the creation of unique organic and inorganic structures with high precision. In urology, the technology has demonstrated potential uses in both patient and clinician education as well as in clinical practice. The 4 major techniques used for 3DP are inkjet printing, extrusion printing, laser sintering, and STL. 3DP is currently being applied to create implantable devices, such as ureteral and urethral stents, as well as inorganic models for surgical planning. Animal studies are already underway for the creation of 3D organic constructs that are intended to replace vital organs, including the bladder, kidneys, and urethra. The goal of bioprinting 3D organic constructs is to provide a personalized solution for organ replacement, alleviating the shortage of suitable transplant organs and associated complications [16]. There are alternative uses for 3DP in different areas of urology along with their potential use, such as the resection planning of genitourinary organs; prostate biopsies; determining detailed and accurate imaging before surgeries, like percutaneous nephrolithotomy; operation decisions on both blunt and sharp traumas; culture models, in order to create organs; and tactile anatomical models for medical students and surgical residents [17]. Notwithstanding the current limitations and the sporadic experiences available in the literature, 3D model technology is perceived as a useful tool for surgical planning, especially in the fields of kidney and prostate cancer, physician education/training, and patient counseling [18]. Despite the promise that 3DP has shown in the medical literature, major barriers exist, apart from the obvious financial burden, for the technology, which is being adopted widely. First, clinicians often lack the technical skills required to segment medical images and print 3D models of their patient’s anatomy. Second, the scarcity of biocompatible materials for printing patient-specific implantable components limits the use of this approach. Third, conventional sterilization via an autoclave requires contact with high temperatures (121–132 °C) and significant pressure, which most 3D-printed materials cannot withstand [19].

When the recent literature was reviewed for urological 3DP, most of the work was related to soft tissue modeling of the kidney [20–32], and few were related to the prostate [31], vesico-urethral anastomosis [33], and sacral neuromodulation [34]. Adams et al. [20] reconstructed detailed anatomical kidney models directly acquired from high-resolution CT data sets of human cadaveric kidneys. CT reconstruction, ultrasound examination, and endoscopy showed that the designed phantom mimics a real kidney’s detailed anatomy and correctly corresponds to the targeted human cadaver’s upper urinary tract. They found that the method was a cost-effective means for obtaining a reproducible and robust model suitable for surgical simulation and training purposes. Glybochko et al. [25] produced personalized 3D-printed models based on CT images of 5 patients with kidney tumors. Next, 5 surgeons took part in a survey in which the utility of CT images versus the 3-dimensional (3D) printed models for presurgical planning was compared. The same surgeons, in a surgical training box, performed a laparoscopic partial nephrectomy training using the developed 3D-printed models. They stated that 3D-printed models allowed one to evaluate the pathological anatomy of tumors more effectively and the high similarity between 3D-printed models and native kidneys contributed to the improvement of the surgical skills necessary for a partial nephrectomy. They added that training on the 3D-printed models also allowed surgeons to determine an optimal surgical maneuver for each patient. 

Atalay et al. investigated the impact of 3D-printed pelvicalyceal system models on residents’ understanding of pelvicalyceal system anatomy [21] and patient information [22] before percutaneous nephrolithotripsy (PCNL). After producing and presenting to the residents 5 patients’ anatomically accurate models of the human renal collecting system, the residents were 86% and 88% better at determining the number of anterior and posterior calyces, respectively, 60% better at understanding the stone location, and 64% better at determining the optimal entry calyx into the collecting system [21]. Similarly they stated that after the 3D-printed model presentation, the patients demonstrated an improvement in their understanding of basic kidney anatomy by 60%, kidney stone position by 50%, the planned surgical procedure by 60%, and the complications related to the surgery by 64% [22]. Bernhard et al. [23] and Wake et al. [31] had similar improvements in patients’ understanding of kidney anatomy and physiology, tumor characteristics, and planned surgical procedures. 

Ghazi et al. [24] produced anatomically correct models of the human pelvicalyceal system using poly-vinyl alcohol hydrogels and 3D-printed injection molds. They assessed the face and content validity of the models with 5 experts (>100 caseload) and 10 novices (<20 caseload). There were significant differences between the novice and expert operative metrics including the mean fluoroscopy time, number of percutaneous access attempts, and number of times the needle was repositioned. The experts achieved better stone clearance with fewer procedural complications. 

Knoedler et al. [27] evaluated the effects of 6 different 3D-printed physical renal models, which were printed from a transparent plastic resin. The normal parenchyma was printed in a clear, translucent plastic with a red hue delineating the suspicious renal lesion, with enhancing masses on the medical trainee characterization, localization, and understanding of renal malignancy. Overall trainee nephrometry score accuracy was significantly improved with the 3D model vs. CT scan. Furthermore, 3 of the 4 components of the nephrometry score (radius, nearness to collecting system, and location) showed significant improvement using the models. 

Lee et al. [28] produced personalized renal models using 3DP methods from the preoperative CT images of a total of 10 patients. In 2 different groups (urologist and student groups), the clinical usefulness of 3D renal models were appraised by answering questionnaires. After application of the 3D renal models, the urologist group gave highly positive responses to the question of the clinical usefulness of the 3D-model in understanding personal human anatomy, preoperative surgical planning, intraoperative tumor localization, planning for further utilization in the future, and clinical benefits in a completely endophytic mass. After the introduction of the 3D-models, the student group located each renal tumor correctly and the rate of correct answers was significantly elevated to 70.0% from 47.3% when they solely interpreted the CT images. The subjective difficulty level in localizing the renal tumor was significantly low when they utilized the 3D models (27% vs. 52% respectively).

Surgeon training in the twenty-first century is subject to a myriad of pressures, including reduced hours available for training and increased threat of litigation against their operating practice. The Halstedian approach of “see one, do one, teach one” has been replaced within surgical training and simulation has become established to enable urology trainees to develop technical and nontechnical skills outside of the operating room. With the primary focus as patient safety and increasing operating skills, “simulation training” encompasses several modalities, including VR and AR. To incorporate simulators into training, models must be carefully designed and evaluated according to certain considerations, ensuring that they address parameters such as face, content, and construct validity [35]. Clements et al. [36] aimed to identify that the changes in simulator usage, and the presence of formal curricula in the wake of technological advances and changes in graduate medical education. Attendees, mostly in their second or third year of residency, were surveyed on the availability and use of laparoscopic/robotic simulators in their program. According to their results, the availability of VR simulators increased from 14% to 60%; however, the frequency of simulator use remained unchanged. There was also a decrease in the percentage of residents who felt that official laparoscopic curricula (93% to 81%) and simulators (82% to 74%) should be involved in resident education. VR simulators were used and assessed in the different surgical procedures and skills in the field of urology, including partial nephrectomy [37, 38], PCA [39], PCNL [40], transurethral resection of bladder tumors [41, 42], transurethral resection of the prostate [43], holmium laser enucleation of the prostate [44], varicocelectomy [45], vesico-urethral anastomosis [46], and ureteroscopic stone extraction skills [47]. 

Hung et al. [37] assessed the face, content, and construct validity of a hybrid platform that contained VR and AR features, and the participants were classified as novice (no surgical training, 15), intermediate (less than 100 robotic cases, 13), and expert (100 or more robotic cases, 14). The experts rated the AR content as realistic and helpful for resident/fellowship training. The experts rated the platform highly for teaching anatomy and operative steps, but moderately for technical skills. Performance in the procedure-specific VR task correlated highly with a porcine model (concurrent validity).

Noureldin et al. [39] studied the competency of urology postgraduate trainees (PGTs) in PCA. When compared with the 21 PGTs without practice, all 5 PGTs who had practiced on the simulator were competent, performed the task with significantly shorter operative and fluoroscopy time, and had significantly higher scores and successful attempts to access renal calyces.

Aside from VR-based simulators, there is a new trend of the development and AR applications use in urology, such as laporoscopic skills [48] and PCNL [49]. There are also few published studies about the feasibility and safety of AR-assisted urological surgery using smart glasses [50, 51]. Bertolo et al. [52] stressed that, based on the existing evidence, they were unable to state that AR improved the outcomes of urological interventions. They thought the major limitation of AR-assisted surgery was inaccuracy in registration, translating into a poor navigation precision. 

## 5. Conclusion

Herein, the following important impacts were achieved with the MedTRain3DModSim project on urology training, both anatomically and surgically.

First, we experienced ‘hybrid anatomy education’, using 3D digital and printed models. When the southeastern European region, which includes Italy, Greece, and Turkey, was examined cadaver donation was limited and less than in the other regions of Europe. The use of printed and/or digital 3D anatomical models in anatomy education provided content and increased the quality of using that content. It also provided variety and diversity to the limited educational materials because of the aforementioned cadaver restrictions. With the experience of our South Korean partner on the utilization of anatomical models in animations and simulations, the anatomical models produced by this project formed a big data set, which was important for producing literal or letter-perfect animations and simulations that were much closer to the real case. 

The second impact was the idea of a health sciences 3D modeling unit. The idea was formed with the support of Mustafa Kemal University after our multiplier event was held in Antakya. In addition, 2 news articles (‘virtual surgery applications’ and ‘surgery with 3D medical printing’) about our project appeared in the national press. That provided positive attention to the subject and conveyed the intellectual outputs of the project to the public. 

The third impact was the integration of a 3D medical modeling system in the medical training curriculum. We tried to reach this goal through our IOs, LTT activities, ME, and dissemination courses. Along with this idea, another important effect of the project was forming a collaboration among the countries related to medical education training and novel 3D medical modeling. We formed a strong network among the participating countries: Greece, Italy, Czech Republic, and South Korea. We made a presentation at an IEEE conference about 3D medical model standardization.

The fourth was the team formation on the 3D medical applications and modeling in Europe, and sharing academic and practical experience with the other countries in Europe. With our website club (Medtrain3Dmodsim Club) and LTT activities, we tried to reach every county in EU and the world. 

The fifth was the identification systematic syllabus on medical training using the new technologies and easy access novel training models like the VR and AR simulators. As a MedTrain3DModsim team, we have been working on systematic curriculum, especially related to urology and general surgery skills. We also contacted associations like the European Association of Urology and International Continence Society for standardization of the surgical skills with 3D models and simulators. 

And finally, the sixth was positively affecting the research, training, and patient care using novel 3D medical applications in daily clinical practice and educational sessions. With this in mind, it is our aim to write a new project about surgical planning with these 3D models and simulations. It was also believed that surgical complications could be decreased if more 3D medical surgical models were used within surgical training. The learning curve of surgical anatomy could be improved with these models depicting correct anatomical plans, proper surgical planning, and increased visualization of solid organ anatomy.

## Acknowledgment

MedTRain3DModsim Erasmus + European Union Project, 2016-1-TR01-KA203-034929, was funded by Ministry of Foreign Affairs, National Agency of Turkey.
